# Down-regulation of *GRP78* in human glaucomatous trabecular meshwork cells

**Published:** 2010-06-17

**Authors:** Fang Chai, Rongjiang Luo, Yiqing Li, Yujing Bai, Yuan He, Yantao Wei, Zhichao Yan, Jian Ge, Yehong Zhuo

**Affiliations:** 1 State Key Laboratory of Ophthalmology, Zhongshan Ophthalmic Center, Sun Yat-sen University, Guangzhou, People’s Republic of China; 2 Department of Ophthalmology in Xi’an No.4 Hospital, Xi’an, People’s Republic of China; 3 Department of Ophthalmology in the First Affiliated Hospital of Sun Yat-sen University, Guangzhou, People’s Republic of China; 4 Department of Ophthalmology in the Second Affiliated Hospital of Xi’an College of Medicine, Xi’an, People’s Republic of China

## Abstract

**Purpose:**

Since the 78 kDa glucose-regulated protein (GRP78) is a key marker of endoplasmic reticulum (ER) stress, we investigated and analyzed *GRP78* expression levels in the trabecular meshwork (TM) by eyes with primary open angle glaucoma (POAG) and normal eyes to understand the role of GRP78 in human TM cells apoptosis.

**Methods:**

Trabecular meshwork cells from POAG patients (GTM) and age-matched non-diseased individuals (NTM) were cultured and treated with or without the following chemical chaperones: tunicamycin (Tm), which induces ER stress, and staurosporine (STS), which induce apoptosis, and dimethylsulfoxide (DMSO) as a control, for 6–24 h. Expression of *GRP78* mRNA and protein was measured using real-time PCR and western blot analysis. The intracellular distribution of GRP78 with myocilin was analyzed using confocal double immunoﬂuorescence.

**Results:**

Both real-time PCR and western blot analysis showed similar results, revealing that *GRP78* mRNA expression and GRP78 protein levels were attenuated in GTM cells compared with NTM cells (65.97±3.8% and 80.49±4.2%, respectively, p<0.05). After exposure to Tm and following ER stress, increased GRP78 protein levels were detected in all cells. However, a low fold change of the protein (2.564 versus 2.710 for a 24 h exposure) and lower cell viability were found in GTM cells compared to NTM cells (p<0.05). Confocal microscopy showed that GRP78 was partly colocalized with myocilin in GTM cells, but less in NTM cells. After Tm and STS treatment, the colocalization of GRP78 with myocilin was found in both NTM and GTM cells.

**Conclusions:**

The authors propose that the down-regulation of *GRP78* plays a role in the degeneration of TM cells in POAG patients, thus providing molecular insights into the pathogenesis of POAG and suggesting that *GRP78* may have the potential to be a target for developing new modalities for ER stress-induced TM cell apoptosis.

## Introduction

Glaucoma, characterized by progressive optic neuropathy, is the second most common cause of blindness in the world [1]. Primary open angle glaucoma (POAG) is the most common form of glaucoma, yet its pathogenesis is still unknown. POAG is generally associated with elevated intraocular pressure (IOP) caused by the abnormal resistance of aqueous outflow through the trabecular meshwork (TM), a specialized tissue lining the outflow pathway of the eye [2,3]. Elevated IOP can lead to progressive neuropathy and retinal ganglion cell (RGC) death in the retina, conditions that result in irreversible vision loss [4-7]. It has been suggested that age- and disease-related changes in TM cells, followed by substitution with extracellular matrix, contributes to an increased resistance to aqueous outflow and causes increased IOP in POAG patients [8-13].

Endoplasmic reticulum (ER) stress modifies the normal folding of protein, resulting in accumulation of anomalous proteins which cause cytotoxicity of a cell’s internal living environment [14,15]. Both in vitro and in vivo data strongly indicated that the accumulation of mutant myocilin in the ER of human trabecular meshwork (HTM) cells is one reason for the development of myocilin-associated glaucoma [16,17]. A possible reason for the inducing of glaucoma by mutant MYOC may be the ability of the stressed ER activated the unfolded protein response (UPR) to then influence the synthesis, folding, and sorting [17-20]. UPR is a signal transduction cascade that perceives and moderates protein-folding stress in the ER caused by physiologic constitution or environmental variation [21]. The 78 kDa glucose-regulated protein (GRP78) is an abundant multi-functional protein that binds to each of the ER stress transducers, including PKR-like ER kinase (PERK),inositol-requiring enzyme 1 (IRE1), and activating transcription factor 6 (ATF6) and serves as a transmitter in alterating of ER homeostasis. These proteins originally generate a cytoprotective signals leading to reduced translation, then improve ER protein folding capacity, and clear misfolded ER proteins [14,15]. Studies performed during the last decade identified GRP78 as a ubiquitous luminal resident protein of the ER that plays a key role in assisting the corrected folding of protein tertiary and quaternary structures. This is defined as chaperoning, and implies the direct binding of GRP78 to the growing chains, with stimulation of its ATPase activity [22,23]. Cytoprotective outputs coexist, along with pro-apoptotic signaling, outweighing this effect in the initial stages of ER stress. The acute UPR allows cells to readjust protein synthesis and chaperone levels to deal with stress. If these steps fail to reestablish homeostasis, IRE1α and ATF6α signals will be attenuated, creating an imbalance where pro-apoptotic output guides the cell toward apoptosis [24].

Recently studies indicated a close connection between GRP78 and ER stress in certain disease processes. The protein can protects cells from ER stress and its induction is crucial for maintaining the viability of cells subjected to a variety of stress treatments [25]. Hayashi et al. [26] found that induction of GRP78 by ischemic preconditioning reduces endoplasmic reticulum stress and prevents delayed neuronal cell death. The overexpression of GRP78 results in reduced apoptosis in Chinese hamster ovary cells [27] and cardiomyocytes [28]. This effect is associated with the formation of the GRP78*-*caspase-7 complex through an ATP-binding domain within GRP78. The role of GRP78 during stress is not limited to quality control of the misfolded proteins, but also includes an increase in Ca^2+^ buffering within the ER lumen. For example, increased levels of GRP78 in HeLa cells leads to appreciable increases of ER Ca^2+^ storage capacity [29].

The role of GRP78 in normal and diseased outﬂow pathways that induces the elevation of intraocular pressure has not been demonstrated. We previously demonstrated that Pro370Leu mutant myocilin attenuated the induction of GRP78 and the phosphorylation of eIF2α [30]. Speciﬁcally, HTM cells expressing Pro370Leu mutant myocilin showed a selective decrease in *GRP78* mRNA compared to wild-type myocilin at the same age. This correlation suggested that insufﬁcient GRP78 levels may contribute to TM cell death.

So, we hypothesize that the increasing levels of GRP78 may be beneﬁcial in preventing POAG associated with myocilin misfolding. However, the overexpression of GRP78 has not yet been studied in TM cells from POAG patients (GTM) and non-diseased individuals (NTM). In this study, we focus on investigating the expression of GRP78 in normal and diseased HTM cells and on studing the relationship between GRP78 and MYOC, which is the common mutant protein in POAG.

## Methods

### Patient information - clinical findings in primary open angle glaucoma

Prior to the surgery, clinical data for each patient were collected, including age, gender, use of prostaglandin analogs, number of argon laser trabeculoplasties and other ocular surgical interventions, type and duration of glaucoma, IOP, and visual acuity. Glaucoma diagnoses were based on careful clinical eye examination, including slit lamp examinations, optical coherence tomography, gonioscopy, fundus photography and visual field examinations. All patients underwent slit lamp examination again the day before surgery. All IOPs in the POAG group exceeded 20 mmHg at the time of surgery. Visual acuity varied from 0.3 to 1.0.

### Tissue procurement and cell culture

Normal human eyes were obtained from the Zhongshan Ophthalmic Center Eye Bank in Guangzhou, China. The procurement of tissue was approved by the IRB Committee at the Sun Yat-sen University in Guangzhou, China. Normal TM cells were derived from eight human donor eyes, without any known ocular diseases, for corneal transplantation, less than 24 h after death. The ages of the donors ranged from 20 to 60 years. After written informed consent, TM specimens from eight POAG patients (15 to 60 years old) were obtained by standard surgical trabeculectomy for therapeutic purposes, less than 1 h after surgery. In brief, the human TM cells were carefully dissected from the anterior segments and the whole corneal layer of the human donor eyes. The explants were placed in 24-well culture plates (Corning Costar, Cambridge, MA) containing Dulbecco’s Modified Eagle Medium (DMEM) which was supplemented with 15% fetal bovine serum, 2 mM L-glutamine, penicillin (100 U/ml), and streptomycin (100 µg/ml). Cells from the TM migrated from the explants within approximately seven days and formed a confluent monolayer in two to five days. Second- or third-passage cells were used for all the experiments described here.

### Tunicamycin (Tm) and staurosporine (STS) treatment

Stock solutions of 1 μM Tm (an inducer of ER stress) and 0.1 μM STS (a well characterized inducer of apoptosis) were dissolved in dimethyl sulfoxide (DMSO) and stored at 4 °C. The NTM and GTM cells were treated with Tm and STS, as mentioned below. All TM cells were divided into a control and treatment groups. Treatment groups were grown in media containing 1μM Tm or 0.1μ mol/L STS for six and 24h to examine GRP78 expression, the others were grown in normal media and received equivalent volumes of DMSO as a control. Morphological changes in the primary cultures were examined using light microscopy.

### Cell viability

A Cell Titer 96® AQueous One Solution cell proliferation assay (Promega, Madison, WI) was used to evaluate cell viability [18]. The NTM and GTM cells were seeded in 96-well plates with an initial density of 5×10^3^ cells per well and were incubated for 24 h (reaching 80% confluence) before the copolymers were added. Then, serum-supplied DMEM was replaced with 200 µl of serum and antibiotic-free DMEM that contained various concentrations (0, 1, 3, 10, 30, and 100 uM) of Tm. After six or 24 h incubation at 37 °C, the media were replaced with fresh serum and antibiotic-free DMEM that contained 20 μl of Cell Titer 96® AQueous One Solution Reagent (MTS). Finally, after 4 h of additional incubation, a micro-plate reader (Bio-Rad Lab Inc., Hercules, CA) measured the absorbance of each well at 570 nm. Cell viability was calculated according to the following equation:

Cell viability (%)=(OD570 [sample]/OD570 [control)×100;

where OD570 (sample) represents the average absorbance of cells treated with media that contain different concentrations of cationic polymer complexes, and OD570 (control) represents the average absorbance of cells treated only with an equal volume of serum-free DMEM.

### Confocal laser scanning microscopy

Cells seeded on polylysine (10 μg/ml)-coated glass chamber slides at a density of 2,000 cells/chamber were washed, fixed in ice-cold 4% paraformaldehyde for 15 min, and permeabilized in 100 mM phosphate buffer containing 0.2% TritonX-100 (Sigma-Aldrich, St. Louis, MO) for 4 min. The cells were then incubated with 5% bovine serum albumin (BSA), immunolabeled with anti-GRP78 (1:500; Santa Cruz Biotechnology, Inc., Santa Cruz, CA) and anti-MYOC (1:500; Santa Cruz Biotechnology, Inc.) at room temperature for 1 h. Normal goat IgG was used instead of anti-GRP78 in some experiments to serve as a negative control. After incubation with the primary antibody, the cells were washed and incubated for 1h with Cy3-anti-GRP78 (1:500; Santa Cruz Biotechnology, Inc.) and FITC-anti-MYOC (1:500; Santa Cruz Biotechnology, Inc.) for 1 h. After additional washes, the cells were mounted using fluorescence mounting medium (Applygen Technologies, Inc., Beijing, China). The staining pattern was visualized using a Zeiss 100M confocal microscope (Carl Zeiss Jena GmbH, Jena, Germany).

### Real-time reverse transcription polymerase chain reaction

To examine *GRP78* mRNA expression, NTM and GTM cells were seeded in 6-well plates at a density of 1.4×10^5^ cells per well. After the cells were incubated for 24h, they were treated in media without fetal bovine serum (FBS; HyClone, Logan, UT) to achieve synchronization. They were then exposed to 1 µM Tm or 0.1µM STS in 10% FBS DF12 for six and 24 h. Total RNA was extracted using an RNeasy Micro Kit (Qiagen Inc., Valencia, CA) according to the manufacturer’s protocol. The total RNA was divided into microtubes, and frozen to −80 ^o^С. The yield and purity of RNA were determined spectrophotometrically at 260 nm, and the cDNA was prepared using the ReverAid^TM^ First Stand cDNA Synthesis Kit (Fermentas Inc., Hanover, MD). A real-time reverse transcription polymerase chain reaction (RT–PCR) procedure was conducted according to the manufacturer’s protocol for the SYBR^®^ Premix Ex TaqTM Kit (Takara Biotechnology, Otsu, Shiga, Japan). Reaction participants were assembled in a 96-well optical reaction plate (Applied Biosystems, Foster City, CA), where each well contained SYBR® Premix Ex Taq™ (2×), 200 nM of forward primer, 200nM of reverse primer, ROX Reference Dye (50×), and cDNA solution, with a total volume of 20 μl. For *GRP78*, the forward primer was 5′-GAC ATC AAG TTC TTG CCG TT-3′, and the reverse primer was 5′-CTC ATA ACA TTT AGG CCA GC-3′. The mRNA level of glyceraldehydes-3-phosphate dehydrogenase (*GAPDH*) was also measured in each sample as an internal control. The forward primer was 5′-GAG TCA ACG GAT TTG GTC GT-3′, and the reverse primer was 5′-CAT GGG TGG AAT CAT ATT GGA-3′. Reactions were performed under the following conditions: 10 min at 95 °C for the initial denaturation, 40 cycles of amplification (5 s at 95 °C), and annealing for 31 s at 60 °C, using the ABI Prism 7000 Sequence Detection System (Applied Biosystems). The threshold cycle (C_t_) values were determined by ABI Prism 7000 Software (Applied Biosystems) and normalized by subtracting the C_t_ *GAPDH* values. All experiments were performed in triplicate, and the relative amount of mRNA of each sample was calculated using the 2-ΔC_t_ method in individual experiments [19].

### Western blot analysis

After treatment, HTM cells were treated with cold phosphate buffered saline (PBS, pH7.4) and lysed in 100 µl of protein extraction reagent (Pierce, Rockford, IL). After boiling for 5 min, the cell lysates were centrifuged at 4 °C for 15 min and the supernatants were collected and stored at −80 °C. Each sample (20 µg) was loaded onto 10% SDS-polyacrylamide gels and electrophoresed at 100 V for 1 h. The resolved proteins were transferred electrically to polyvinylidene difluoride (PVDF) membranes (Invitrogen, Carlsbad, CA) and incubated with 0.5% skim milk in tris-buffered saline containing 0.05% Tween-20 (TPBS) for 1.5 h. The membrane was probed with goat polyclonal GRP78 antibody (1:1,000 final dilution; Santa Cruz Biotechnology, Inc.) and developed with Phototope-HRP rabbit anti-goat polyclonal antibody (1:1,000 final dilution; Santa Cruz Biotechnology, Inc.). Blotting signals were detected by chemiluminescence reagents using SuperECL Plus (Applygen Technologies, Inc.) following the manufacturer’s instructions. The amount of β-actin protein in each sample was also measured as an internal control.

### Statistical analysis

GraphPad Prizm 5 software (GraphPad Software Inc., San Diego, CA) and SPSS version 13.0 for windows (SPSS Science Inc., Chicago,IL) statistical software packages were used. The relative amount of mRNA in each sample was compared with untreated NTM and calculated using the 2-ΔC_t_ method in individual experiments. After quantification of western blot data, the mean±SD are shown. *T*-test analysis was used for data obtained from NTM and GTM cells. A one-way ANOVA test was performed for comparisons among multiple groups, and statistical significance was set at p<0.05. All assays were performed using at least three separate experiments in triplicate, and data were expressed as mean±SD in comparisons with untreated cells (controls).

## Results

### Morphology changes in HTM cells treated with Tm and STS

**Figure 1:**
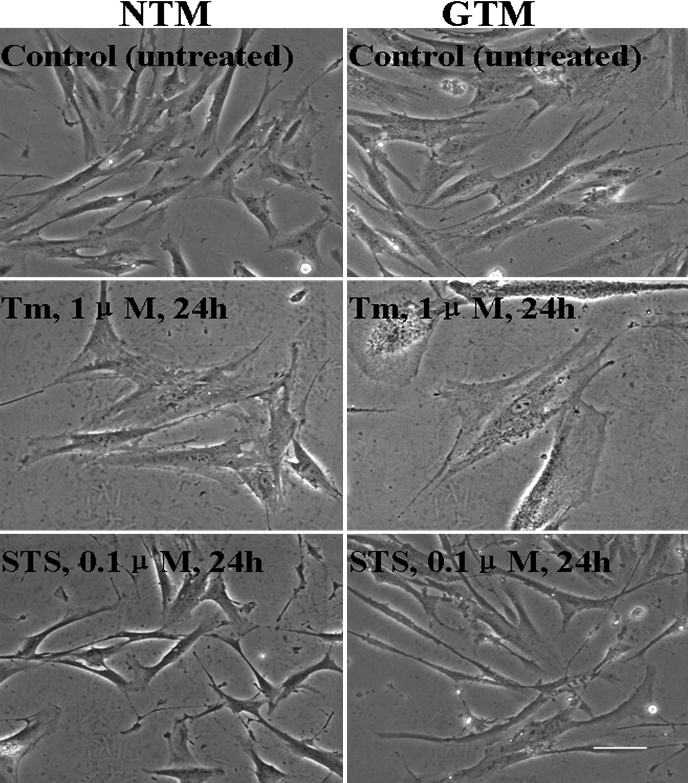
Morphology of TM cells before and after Tm or STS treatment. NTM and GTM cells were obtained and cultured using identical culture protocols. GTM cells were slightly larger compared to NTM cells. The morphology of the cells became thin and slender after treatment with 1 μM Tm or 0.1 μM STS for 24 h as compared to untreated controls, especially in the STS-treated group. Gross morphological alterations (cell shrinkage, low density) were apparent at 24 h after STS treatment. Scale bar=30 μm.

Apoptosis was induced in TM cells using Tm (1 uM) or STS (0.1 uM), and morphological changes were assessed using light microscopy. The NTM and GTM cells were cultured and characterized as previously described [22,23]. As shown in Figure 1, GTM cells appeared to be larger and more irregular in shape compared to NTM cells. After being incubated with Tm (1 μM) or STS (0.1 μM) for 24 h and using phase-contrast light microscope, it was found that the cells in the treatment groups shrunk compared to those in the control group (Figure 1), especially in the STS-treated group. At the same time, the density of NTM and GTM cells was decreased after being treated with the drugs.

### Detecting cytotoxicity of Tm and STS

**Figure 2:**
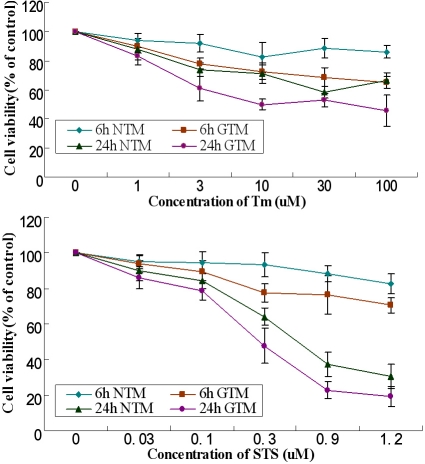
Determination of dynamic alteration of cell proliferation in activated HTM cells after exposure to Tm or STS by MTS assay. Data were presented as the percentage of viable cells compared with untreated (control) cells (mean±SD, n=6).

A tetrazolium-based viability assay, based on the bioreduction of the MTS reagent into formazan by living cells, was used to study the cytotoxicity of Tm and STS. The TM cells exhibited cell viability of more than 80% at concentrations of 1 uM, and the number of viable cells was significantly higher than those treated with STS at concentrations of 0.1 μg/ml and above (Figure 2).

### Attenuated *GRP78* expression in GTM compared to NTM cells

**Figure 3:**
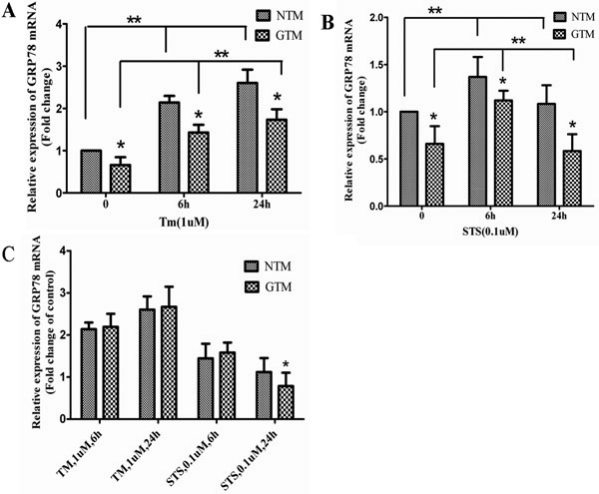
Both NTM and GTM cells were treated as indicated, and normalized *GRP78* mRNA levels were measured using real time PCR and were shown relative to levels in untreated cells. All experiments were performed in triplicate, and the relative amount of mRNA in each sample was calculated using the 2-ΔC_t_ method in individual experiments. Values represent the means±SDs, ANOVA test, the double asterisk indicates a p<0.05; Student’s *t*-test, and the asterisk indicates a p<0.05.

**Figure 4:**
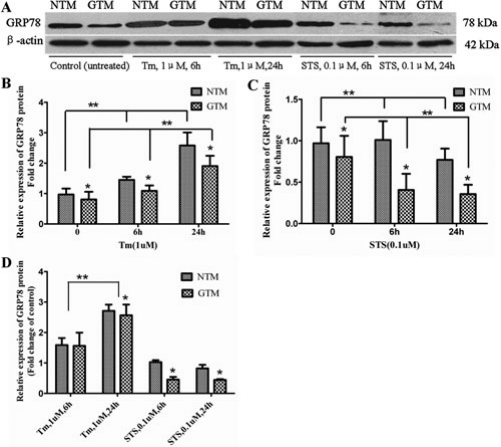
Normalized GRP78 protein levels were measured in cells (treated with Tm or STS) using western blot analysis and were shown relative to levels in no treatment cells. Meanwhile, the expression peak of GRP78 protein appeared at 24 h after treatment with Tm. After exposure to Tm and following ER stress, increased GRP78 protein levels were detected in all cells, however, a lower fold change of the protein (2.564 versus 2.710 for 24 h exposure) was found in GTM cells compared to NTM cells (p<0.05). Error bars represent SDs from three individual experiments, ANOVA test, the double asterisk indicates a p<0.05; Student’s *t*-test, and the asterisk indicates a p<0.05.

A real-time PCR assay revealed that *GRP78* mRNA in GTM cells exhibited a significant decrease (65.97±3.8%, mean±SD, n=3, p<0.05) compared to NTM cells (Table 1, Figure 3). The relative expression developed into 66.89±1.7%, 66.54±1.9% when cells were treated with Tm for six or 24 h (Table 1, Figure 3A) and 79.10±1.6%, 52.10±3.1% with STS for six or 24 h (Table 1, Figure 3B). Based on western blot analysis, the expression of GRP78 protein was attenuated (80.49±4.2%, mean±SD, n=3, p<0.05) compared with NTM cells (Table 2, Figure 4). The relative expression became 72.78±2.1%, 71.54±2.2% when cells were treated with Tm (Table 2, Figure 4B) and 38.96±3.2%, 44.89±2.4% when cells were treated with STS (Table 2, Figure 4C) for six h and 24 h, respectively. β-actin was used as an internal loading control.

**Table 1:** Relative expression of *GRP78* mRNA.

** **	**Treatment**				
**Group**	**Control**	**Tm, 1 μM, 6 h**	**Tm, 1 μM, 24 h**	**STS, 0.1 μM, 6 h**	**STS, 0.1 μM, 24 h**
NTM (mean±SD) n=3	1.000±0.000	2.138±0.032	2.604±0.062	1.369±0.038	1.119±0.061
GTM (mean±SD) n=3	0.660±0.038	1.430±0.037	1.732±0.049	1.083±0.022	0.583±0.035
GTM/NTM	65.97±3.8%*	66.89±1.7%*	66.54±1.9%*	79.10±1.6%*	52.10±3.1%*

The asterisk indicates a p<0.05.

**Table 2:** Relative expression of GRP78 protein.

** **	**Treatment**				
**Group**	**Control**	**Tm, 1 uM, 6 h**	**Tm, 1 uM, 24 h**	**STS, 0.1 uM, 6 h**	**STS, 0.1 uM, 24 h**
NTM (mean±SD) n=3	1.000±0.033	1.495±0.018	2.664±0.074	1.042±0.038	0.793±0.023
GTM (mean±SD) n=3	0.805±0.042	1.088±0.031	1.906±0.058	0.406±0.033	0.356±0.019
GTM/NTM	80.49±4.2%*	72.78±2.1%*	71.54±2.2%*	38.96±3.2%*	44.89±2.4%*

The asterisk indicates a p<0.05.

### Tm upregulated GRP78 expression in a time-dependent manner, while STS decreased GRP78 protein expression in GTM cells

The upregulation of GRP78 expression in human TM cells was obvious when the cells were treated with Tm(Figure 3A, Figure 4A,B). Induction of GRP78 was evident after 6 h of treatment and continued to increase . Maximal induction of *GRP78* in TM cells was about 2.6 fold at 24 h after treatments with Tm (Table 3 and Table 4, p<0.05). There was a significant difference between six h and 24 h of Tm treatment (p<0.05). After exposure to Tm and following ER stress, increased GRP78 protein levels were detected in all cells. However, a lower fold change of the protein (2.367 versus 2.581 for 24 h exposure) was found in GTM cells compared to NTM cells (p<0.05). Staurosporine is an inducer of apoptosis. After being treated with STS, the expression of GRP78 protein decreased (Table 4, Figure 4) in GTM cells compared to NTM cells (p<0.05). No significant difference was detected between six h and 24 h of treatment.

**Table 3:** Relative *GRP78* mRNA expression of control.

** **	**Treatment**			
**Group**	**Tm, 1uM, 6h**	**Tm, 1uM, 24h**	**STS, 0.1uM, 6h**	**STS, 0.1uM, 24h**
NTM	2.138±0.032	2.604±0.062	1.369±0.038	1.119±0.061
GTM	2.16±0.056	2.624±0.074	1.636±0.032	0.881±0.053
GTM/NTM	1.010±0.056	1.008±0.026	1.195±0.018	0.787±0.053*

The asterisk indicates a p<0.05.

**Table 4:** Relative GRP78 protein expression of control.

** **	**Treatment**			
**Group**	**Tm, 1 uM, 6 h**	**Tm, 1 uM, 24 h**	**STS, 0.1 uM, 6 h**	**STS, 0.1 uM, 24 h**
NTM	1.587±0.023	2.710±0.021	1.026±0.064	0.821±0.012
GTM	1.562±0.044	2.564±0.035	0.456±0.009	0.444±0.003
GTM/NTM	0.984±0.024	0.946±0.051*	0.444±0.015*	0.549±0.032*

The asterisk indicates a p<0.05.

### Colocalization of GRP78 and myocilin in TM cells

**Figure 5:**
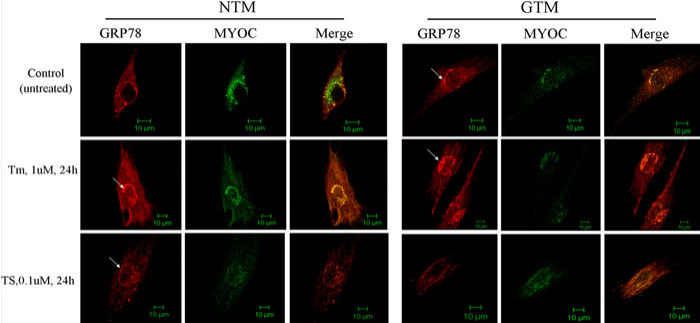
GRP78 and myocilin (MYOC) were expressed in both NTM and GTM cells, and exhibited a diffused distribution throughout the cytoplasm in NTM cells. The expression pattern of GRP78 and MYOC was most overlapping in GTM and treated NTM cells. Perinuclear aggregation of GRP78 expression was observed in GTM and treated NTM cells (white arrow). The Tm slightly upregulated GRP78 expression and STS down-regulated it, while the expression of MYOC did not show much difference. Scale bar=10 μm.

The expression of GRP78 was detected in both NTM and GTM cells (Figure 5). Immunostaining of GRP78 and confocal microscopy demonstrated granular and diffused distribution throughout the cytoplasm in NTM cells. The GRP 78 and myocilin were partly overlapped in the NTM cells, while the overlap range turned into total cytoplasm in GTM and treated NTM cells. Perinuclear aggregation of GRP78 expression was observed in GTM cells (control, Tm-treated group) and treated NTM cells (white arrow).

## Discussion

A decreased number of TM cells was reported as a clinical feature in patients with POAG. Apoptosis has been suggested as being one of the mechanisms by which TM cells die in glaucoma [9,31,32]. The direct molecular mechanism on how TM cell death would lead to an increase in aqueous outﬂow resistance is unclear, but it is more than likely that a severe impairment of TM homeostasis caused by TM cell death should critically impair the main function of the TM. The 78 kDa glucose-regulated protein is involved in polypeptide translocation across the ER membrane and also acts as an apoptotic regulator by protecting the host cell against ER stress-induced cell death. However, the mechanism by which GRP78 exerts its cytoprotective effect is still undetermined. Here, we provide evidence that GRP78 expression is down-regulated in GTM cells compared to NTM cells, that GRP78 can exist as an ER transmembrane protein, and that it can co-localize with myocilin. This was confirmed using fluorescence microscopy.

Studies in cultured HTM cells have suggested that the UPR in response to aggregation of MYOC would represent one disease mechanism in the pathogenesis of intraocular pressure and glaucoma [16,17]. Oxidative stress, glyco-starvation, calcium homeostasis, and misfolded protein accumulation can all lead to ER stress and activation of UPR [15]. UPR elicits paradoxical outputs, inducing cytoprotective functions that re-establish homeostasis and cell destroying functions that promote apoptosis. When these adaptive responses are not sufficient to relieve ER stress, apoptosis is induced [33]. Cytoprotective outputs will outweigh pro-apoptotic factors at this point, which would be helped by the relatively longer mRNA and protein half-lives of factors such as GRP78 [34]. Due to the anti-apoptotic property of GRP78, its induction has been reported as a pro-survival factor for cells undergoing ER stress [26,35].

Overexpression of GRP78 is more necessary needed in cells undergoing physiologic or pathological stress. Previous studies demonstrated that stress may cause expression of GRP78, and that the increase appears to be important for the survival of cancer cells [36-38].The mechanism by which GRP78 overexpression contributes to cancer proliferation is linked to its ability to inactivate pro-apoptotic pathways and activate pro-survival pathways for cancer cells [35]. Reddy et al. [39] provided evidence that specific GRP78 overexpression is sufficient to confer drug resistance in at least two cell types, and that the protective effect of GRP78 is dependent on a functional ATP-binding domain. Our results show that ER stress chaperone GRP78 presents a low level of both mRNA and protein in GTM cells compared with NTM cells. This down-regulation of the ER stress response might disrupt the protective mechanism, thus increasing the vulnerability of GTM cells to ER stress. This correlation suggests that insufﬁcient GRP78 levels contribute to the loss of the cytoprotective function, leading to the death of TM cells.

Low expression of GRP78 alters the susceptibility of TM cells to damage stimulus. Two signaling pathways are proposed to induce expression of GRP78: one responsive to the disturbance of intracellular calcium homeostasis, the other to the inhibition of protein glycosylation [38]. GRP78 are Ca^2+^-binding proteins in the ER, the intracellular Ca^2+^ levels have been implicated as potent regulators in the expression of the GRP78. Moreover, GRP78 is also shown to prevent apoptosis that arises from disturbance of intracellular Ca^2+^, suggesting a critical role of GRP78 in regulating cellular Ca^2+^ homeostasis [40]. He et al. [41] found that POAG patients’ TM cells have a lower ATP level and ΔΨm compared to non-diseased TM cells. The possible mechanism for attenuated GRP78 expression is that it lowers the ER calcium level by releasing calcium stores, which affects protein secretion and causes accumulation of protein in the ER [42,43].

It has been found that GRP78 is located in the cytoplasm and associated with caspase-7 [39]. In cooperation with other chaperones, Hsp70 stabilizes pre-existing proteins against aggregation and mediates the folding of newly translated polypeptides in the cytosol and within organelles. There is also ample evidence that myocilin is a secreted protein; an additional or exclusive intracellular function that has been investigated by various authors [44-46]. In our study, GRP78 expression displayed a diffused distribution throughout the cytoplasm. The overlap of GRP78 staining with the myocilin apparatus also indicates its association with secretory vesicles. Such interference would vary in type and extent according to the type of mutation present, but even a relatively slight interference in endocytoplasmic reticulum function would likely decrease cell viability.

Interestingly, O’Brien’s [47] original observation that myocilin is localized to the golgi in human Schlemm’s canal endothelial cells might also be related to our ﬁndings. What does localization of the GRP78 protein to myocilin mean? The endocytoplasmic reticulum is the site within where glycosylation of proteins occurs, consistent with evidence that myocilin is glycosylated before it is secreted. The mutant MYOC protein induces ER stress and the resultant UPR induces apoptosis in TM cells, which then leads to increased resistance to aqueous humor outflow, elevated IOP, and, ultimately, glaucoma [48].

A growing body of evidence suggests that ER stress causes a buildup of unfolded proteins within the ER, where molecular chaperones (typically GRP78 and PDIs) greatly increased, while the synthesis of new proteins is inhibited [49,50]. Current studies highlight the importance of GRP78 as an anti-apoptotic protein and shed new light on the mechanisms underlying the relationship between ER stress [51,52], UPR, and programmed cell death. The results could have broad implications for our understanding of the mechanism by which cells couple ER stress to the cell death program and the mechanism by which the associated decision between apoptosis inhibition and activation is controlled. Additionally, the results of the present study demonstrate that the induced GRP78 localizes to mitochondria in response to ER stress [53]. Such a mechanism could be a factor in the best-documented evidence of the cellular pathology of the outﬂow pathway and the loss of TM cells in patients with POAG [30]. The correlation between the intracellular myocilin of GRP78 and its possible associations with other proteins has yet to be investigated.

In summary, we found that GRP78 was down-regulated in GTM cells compared to NTM cells, even when treated with an ER stress inducer. Myocilin protein was partly colocalized with GRP78 in TM cells. Nevertheless, the possible mechanism and biologic function of GRP78 in cellular protection warrants further study, the results of which may confirm the idea that such correlation may occur due to GRP78 inactivation, thus helping to specify the diverse functions of GRP78 in response to ER stress.
